# Generation of donor-specific Tr1 cells to be used after kidney transplantation and definition of the timing of their in vivo infusion in the presence of immunosuppression

**DOI:** 10.1186/s12967-017-1133-8

**Published:** 2017-02-21

**Authors:** Bechara Mfarrej, Eleonora Tresoldi, Angela Stabilini, Alessia Paganelli, Rossana Caldara, Antonio Secchi, Manuela Battaglia

**Affiliations:** 1 Diabetes Research Institute (DRI), IRCCS San Raffaele Scientific Institute, Via Olgettina 58, Milan, Italy; 2 Department of Internal Medicine, Transplantation Medicine, San Raffaele Hospital, Milan, Italy; 3 Human T Cell Laboratory, Saint Vincent’s Institute of Medical Research, Melbourne, Australia

**Keywords:** Tolerance induction, Cell therapy, Kidney transplantation, Clinical-grade compatible protocol

## Abstract

**Background:**

Operational tolerance is an alternative to lifelong immunosuppression after transplantation. One strategy to achieve tolerance is by T regulatory cells. Safety and feasibility of a T regulatory type 1 (Tr1)-cell—based therapy to prevent graft versus host disease in patients with hematological malignancies has been already proven. We are now planning to perform a Tr1-cell—based therapy after kidney transplantation.

**Methods:**

Upon tailoring the lab-grade protocol to patients on dialysis, aims of the current work were to develop a clinical-grade compatible protocol to generate a donor-specific Tr1-cell—enriched medicinal product (named T_10_ cells) and to test the Tr1-cell sensitivity to standard immunosuppression in vivo to define the best timing of cell infusion.

**Results:**

We developed a medicinal product that was enriched in Tr1 cells, anergic to donor-cell stimulation, able to suppress proliferation upon donor- but not third-party stimulation in vitro, and stable upon cryopreservation. The protocol was reproducible upon up scaling to leukapheresis from patients on dialysis and was effective in yielding the expected number of T_10_ cells necessary for the planned infusions. The tolerogenic gene signature of circulating Tr1 cells was minimally compromised in kidney transplant recipients under standard immunosuppression and it eventually started to recover 36 weeks post-transplantation, providing rationale for selecting the timings of the cell infusions.

**Conclusions:**

These data provide solid ground for proceeding with the trial and establish robust rationale for defining the correct timing of cell infusion during concomitant immunosuppressive treatment.

**Electronic supplementary material:**

The online version of this article (doi:10.1186/s12967-017-1133-8) contains supplementary material, which is available to authorized users.

## Background

Circulating T regulatory type 1 (Tr1) cells with an alloantigen-specific regulatory function have been consistently associated with operational tolerance after transplantation [1]. Alloantigen-specific Tr1 cells can be induced in vitro in the presence of exogenous IL-10 or by tolerogenic IL-10-producing dendritic cells (DC-10) and they are hyporesponsive (anergic) to the alloantigen used for their generation [2, 3]. These IL-10-anergized T cells have been tested as medicinal product in a proof-of-concept trial in patients undergoing haploidentical hematopoietic stem cell transplantation (HSCT) to provide immune reconstitution in the absence of severe graft versus host disease (G*v*HD) (the ALT-TEN trial) [4].

The ONE Study—a European Commission FP7-funded consortium-aims to test several distinct haematopoietic immunoregulatory cells as therapies after kidney transplantation from living donors by initiating a series of independent clinical trials based on the same general design [5]. Our group participates in this consortium to test donor-specific Tr1 cells. The ALT-TEN trial already performed was certainly instrumental although the know-how that was developed in this first clinical experience did not necessarily allow performing The ONE Study faster and more efficiently. We overcame the first hurdle of tailoring the Tr1-cell generation protocol to patients on dialysis, yet only at a lab-scale [6]. In this study, we aimed at: (1) defining a reproducible and clinical-grade compatible protocol for the generation of a Tr1-cell—enriched medicinal product for kidney transplant recipients, (2) characterizing the final cell product, and (3) testing the sensitivity of circulating Tr1 cells to immunosuppressive therapy to determine the ideal timing of the medicinal product infusion.

## Methods

### Healthy donors and patients

Peripheral blood, buffy coat or leukapheresis were obtained from healthy donors or renal transplant recipients enrolled in The ONE Study Reference Group Trial (i.e., control group in which patients were treated with standard immunosuppressive therapy) (NCT01656135) after written informed consent in accordance with the Declaration of Helsinki under the protocol approved by the San Raffaele Hospital’s Ethics Committee (IRB #OSR-TheOne).

### Generation and characterization of dendritic cells

Peripheral blood mononuclear cells (PBMC) were isolated by density-gradient centrifugation on Lymphoprep (Axis-Shield, Oslo, Norway). IL-10-producing dendritic cells (DC-10) and mature DC (mDC) were generated from healthy donors [7]. Monocytes were isolated by harvesting the adherent fraction of PBMC or by selection with CD14^+^ microbeads using the AutoMACS system (Miltenyi Biotec, Bergisch Gladbach, Germany) and following manufacturer’s instructions. Monocytes were cultured with 10 ng/ml rhIL-4 (GMP-grade, Miltenyi Biotec) and 100 ng/ml rhGM-CSF (GMP-grade, Miltenyi Biotec) for 7 days in the presence (DC-10) or absence (mDC) of 10 ng/ml rhIL-10 (GMP-grade, CellGenix GmbH, Freiburg, Germany). The culture medium was supplemented with GMP-grade fetal bovine serum (FBS) (Lonza, Basel, Switzerland) or GMP-grade human AB sera (Lonza). mDC were matured during the last 2 days of culture with 5 µg/ml of rMPL-A (GMP-grade, Invivogen, Toulouse, France). At the end of the 7-day cultures, DC-10 and mDC were harvested and irradiated at 60 Gy with a Cs137 source Biobeam 2000 irradiator (Gamma-Service Medical GmbH, Leipzig, Germany). DC-10 yield was measured as: 100× [no. of generated DC-10 cells/no. of plated cells].

Supernatants were collected 48 h after culturing DC-10 in the presence or absence of stimulation by lipopolysaccharide (LPS) from *Escherichia coli* (5μg/ml, Sigma Chemicals, St. Louis, MO). IL-10 released into the supernatant was quantified by ELISA (BD Pharmingen, San Diego, CA). The detection limit of IL-10 was 15 pg/ml.

### Generation and characterization of Tr1- cell enriched product: T_10_ cells

**Fig. 1:**
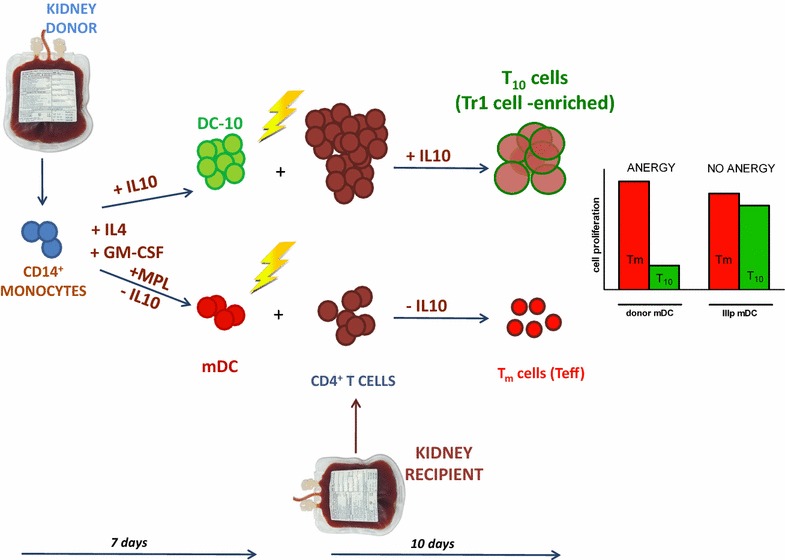
Graphical representation of the protocol for generating T_10_ cells to be used in kidney transplanted patients. CD14^+^ cells are selected from the kidney donor leukapheresis and cultured with GM-CSF and IL-4 in the presence (DC-10) or absence (mDC) of IL-10. At day 5, mDC are activated with monophosphoril MPL. Upon harvest at day 7 of culture, DC-10 and mDC are irradiated and co-cultured for 10 days with CD4^+^ T cells selected from the recipient leukapheresis and exogenous IL-10 to generate T_10_ cells. mDC cultured with CD4^+^ T cells without IL-10 generate the control Tm cell population. T_10_ cells are donor-specific anergic CD4^+^ T cells, yet they respond to third party mDC stimulation (IIIp mDC) similar to the control population as shown by the representative cell-proliferation *bars* (simulating what one should expect when measuring T_10_ cell proliferation toward donor mDC or toward third party unrelated mDC)

CD4^+^ T cells were isolated from donors different from those used to generate DC by CD4^+^ microbeads using the AutoMACS system (Miltenyi Biotec) following manufacturer’s instructions. Purified CD4^+^ T cells were cultured with irradiated allogeneic DC-10 or mDC (10:1 ratio) in the presence or absence of exogenous rhIL-10 (10 ng/ml) for 10 days to generate T_10_ or control Tm cells, respectively (Fig. 1) [8]. T_10_-cell yield was measured as: 100× [no. of T_10_ cells generated/no. CD4^+^ T cells plated]. To test the generation of donor-specific anergic T cells, T_10_ and Tm cells were cultured with the original-donor mDC (previously frozen) and cell proliferation was monitored via ^3^H-thymidine (PerkinElmer, Waltham, MA, USA) incorporation (counts per min, cpm) in the last 16–18 h of a 3-day culture. Anergy was calculated as: cpm [(T_10_ + mDC)/(Tm + mDC)] ×100. Supernatants were collected before ^3^H-thymidine addition and quantification of IFNγ or IL-10 by ELISA (BD Pharmingen) was performed. The detection limit of IFN-γ was 15 pg/ml.

Ability of T_10_ cells to suppress the proliferation of autologous CD4^+^ T cells upon donor or third party mDC stimulation was assessed by ^3^H-thymidine incorporation in the last 16–18 h of a 5-day culture.

### Flow cytometry

The immune phenotype of in vitro generated DC, T_10_ and Tm cells was tested by flow cytometry as previously described [8]. The TCR Vβ repertoire was determined with the IOTest^®^ Beta Mark TCR V beta Repertoire Kit (Beckman Coulter, Inc, Brea, CA, USA) following manufacturer’s instructions.

Cells were analyzed with the BD FACS Canto II (Beckton Dickinson, San Jose, CA, USA) within few hours after staining. Data was analyzed using FCS 3.0 (DeNovo Instruments, Los Angeles, CA, USA).

### Dual IFNγ/IL-10 ELISPOT

Dual IFNγ/IL-10 ELISPOT (Diaclone, Besancon, France) was performed according to manufacturer’s instructions with a slight modification: visualization of IL-10 was performed using Vector Blue Alkaline Phosphatase substrate kit (Vector Labs, Burlingame, CA, USA) and the A.EL.VIS 4-Plate ELISPOT Reader (A.EL.VIS GmbH, Hannover, Germany) was used. Analysis was performed using ImageJ (version 1.48, NIH, USA) to quantify IFNγ-producing cells (red spots), IL-10-producing cells (blue spots) or dual IFNγ/IL-10-producing cells (purple spots).

### Transcript analysis of purified Tr1 cells

Peripheral blood was collected and PBMC were frozen from patients enrolled in The ONE Study Reference Group Trial at our center at the following time points: 4-weeks pre-transplant, 8- 36- and 60-weeks post-transplant. PBMC were thawed and Tr1-cell sorting was performed using MoFlo Legacy Cell Sorter (Beckman Coulter, Indianapolis, IN, USA). To verify the expression of anti-inflammatory genes characteristic of Tr1 cells (as previously described [1]) and pro-inflammatory genes characteristic of T effector cells (i.e., *il*-*17a, il*-*1b, tnf, il*-*6* and *ifnγ*), QuantiGene 15-plex assay (Affymetrix, Santa Clara, CA, USA) was performed following manufacturer’s instructions. Mean fluorescence intensity from the measured beads per gene was reported using Bio-Plex 200 system (BioRad Laboratories, Hercules, CA, USA). Probe set information is provided in Additional file 1.

### Statistical analysis

Comparisons between groups were performed using Student’s t test, Mann–Whitney test or Wilcoxon matched pairs test depending on the experiments. For all analyses, a two-tailed p value ≤0.05 was considered significant. Comparison of variances was performed using the F-test. Statistical analyses were performed using GraphPad Prism version 6.0 (GraphPad Software, San Diego, CA, USA).

## Results

### DC-10 generation in compliance with clinical-grade manufacturing

**Fig. 2:**
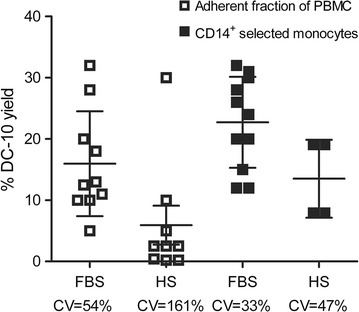
CD14^+^ bead-selected monocytes cultured in FBS-supplemented medium represent an optimal clinical-grade compatible approach for DC-10 generation. Adherent fraction of PBMC (*empty square*) or CD14^+^ selected monocytes (*filled square*) were cultured in FBS- or HS-supplemented medium for 7 days to generate tolerogenic Tr1-cell-inducing DC-10. DC-10 yield is shown, measured as: 100× [no. of generated DC-10 cells/no. of plated cells]. *One square* represents one experiment, *lines* represent mean value of each data set ± SD. Coefficient of variation (CV) is shown for each data set

**Fig. 3:**
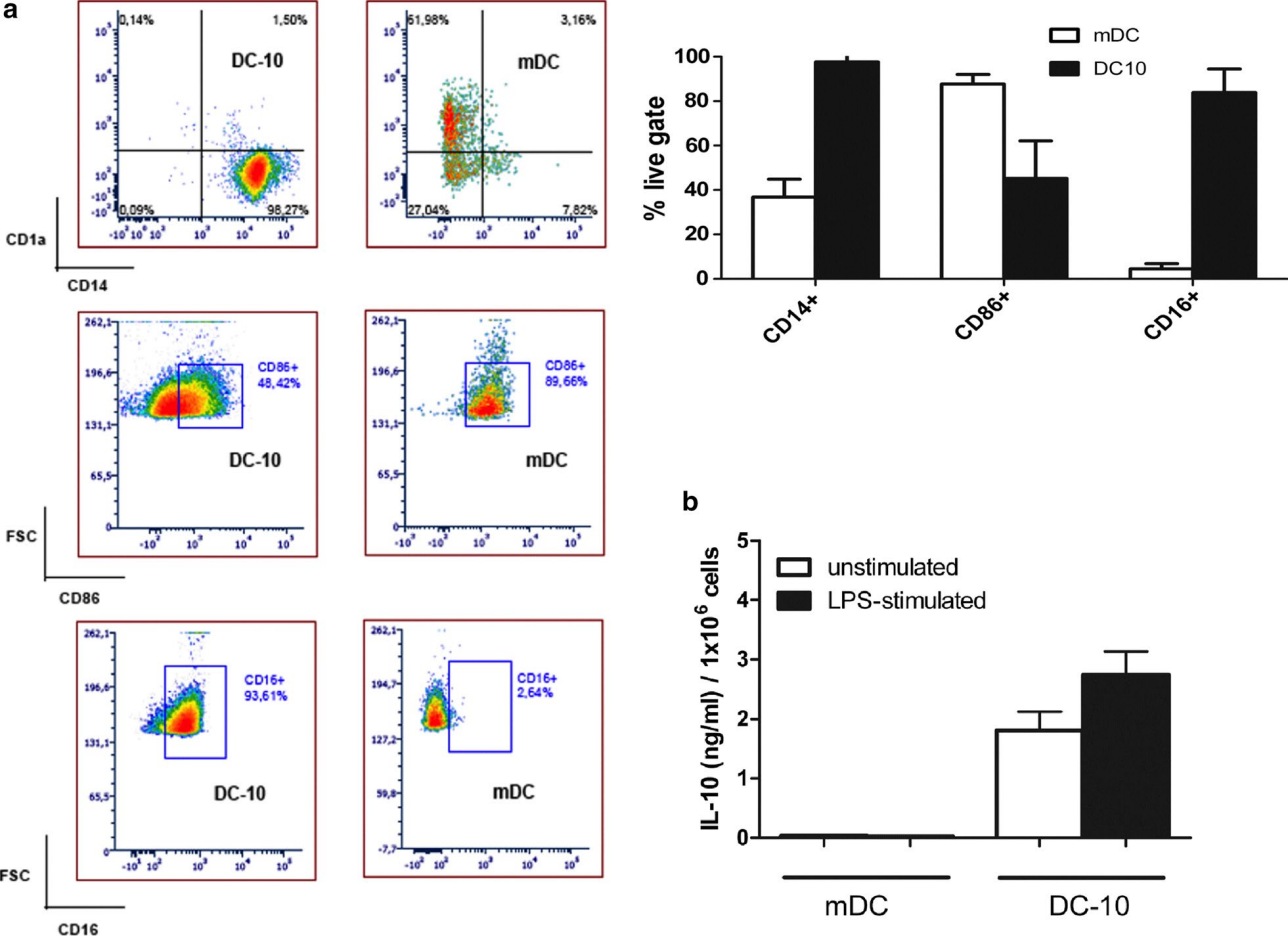
DC-10 have a tolerogenic phenotype and produce IL-10. One representative (out of eight) flow cytometry staining of DC-10 cells and control mDC gated on live cells. Percentages of CD14^+^ cells (*upper left panel* **a**), CD86^+^ cells (*middle left panel* **a**) and CD16^+^ cells (*lower left panel* **a**) are shown. *Bars* represent mean value of each data set ± SD for DC-10 and mDC (*upper right panel* **a**). *Bars* represent mean value of IL-10 (quantified by ELISA) ±SD secreted by DC-10 or mDC upon 48-h of culture in the presence or absence of LPS stimulation (**b**)

The generation of donor-specific Tr1 cells is contingent to the production of donor-derived DC-10 [7]. The first step in defining a clinical-grade compatible protocol was therefore establishing an efficient and reproducible method for DC-10 generation. Based on the MLR/DC-10 protocol used in the ALT-TEN trial [7], DC-10 were generated from the PBMC adherent fraction in medium supplemented with fetal bovine serum (FBS) in wells. Due to the availability of GMP-compatible human serum (HS) and guidelines from the regulatory authorities on the use of bovine-derived sera (EMEA/CHMP Guideline on the use of bovine serum in the manufacture of human biological medicinal products—original version EMA/CPMP/BWP/1793/02, and revised version EMA/CHMP/BWP/457920/2012 rev 1), we compared FBS- and HS-supplemented medium during DC-10 generation. The same batch of FBS was used throughout the experiments described throughout the manuscript. Alongside a certificate of analysis, we confirm the availability of a TSA Certificate of Suitability, issued by EDQM via the process of Certification of Suitability of Monographs of the European Pharmacopoeia. DC-10 recovery was better when medium supplemented with FBS was used, yet the intra-experiment variability—determined by the coefficients of variation (CV)—was high (Fig. 2). This high variability could be attributed to the non-specific monocyte selection method, which leads to unpredictable monocyte recovery [9]. Thus, provided methods for clinical-grade monocyte selection are available, monocytes were purified by CD14^+^ magnetic beads and selected on magnetic columns [10]. DC-10 recovery was less variable upon starting from bead-selected monocytes than from adherence and FBS was necessary to generate sufficient DC-10 numbers (as shown in Fig. 2 and also supported by our unpublished results generated from independent unrelated experiments). DC-10 generated from purified CD14^+^ monocytes in FBS-supplemented medium had the anticipated phenotype (i.e, CD14^+^CD86^+^CD16^+^) and they produced IL-10, either at steady state or upon LPS activation (Fig. 3) [7]. Based on these data, we concluded that peripheral blood monocyte selection by CD14^+^ magnetic beads and culture media supplemented with FBS is the optimal clinical-grade compatible approach for DC-10 generation. This conclusion was drawn based on the limited number of DC-10 generated with HS that was incompatible with the clinical need. In addition, DC-10 will be irradiated and kept in culture with recipient CD4^+^ T cells for 10 additional days in the absence of any bovine-derived products.

### T_10_ cell generation in compliance with clinical-grade manufacturing

A protocol for the generation of cell products to be infused into patients has to be solid and highly reproducible to have the highest chance to be used in clinical trials. Accordingly, buffy coats from eight healthy donors were used for DC-10 generation and buffy coats from eight more donors were used for the isolation of CD4^+^ T cells. Flasks were used as clinical-grade compatible culture containers to generate T_10_ cells.

Monocytes isolated from buffy coats with lab-grade magnetic columns (AutoMACS-Miltenyi) had a mean purity of 95 ± 3% (mean ± SD). DC-10 yield in flasks (13 ± 6, mean ± SD) was, as expected, lower than that in wells (23 ± 7, mean ± SD) (Table 1) but all DC-10 preparations in flasks had a tolerogenic phenotype (Fig. 3).

**Table 1:** Protocol reproducibility with the optimized clinical-grade compatible conditions using buffy coats from eight donor pairs

	CD14^+^ purity (%)	DC-10 yield (%)	CD4^+^ purity (%)	T_10_-cell yield (%)	Donor-specific anergy (%)
Mean	95	13	98	53	80
SD	3	6	1	31	10
Range	90–99	4–21	97–99	17–123	63–94
CV	3.3	47.3	0.9	59.0	14.6

*DC*-*10* tolerogenic dendritic cells, *SD* standard deviation, *CV* coefficient of variation

CD4^+^ T cells isolated from buffy coats with lab-grade magnetic columns had a mean purity of 98 ± 1% (mean ± SD). Average T_10_-cell yield after 10 days of co-culture with allogeneic DC-10 was 53 ± 31% (mean ± SD) and T_10_-cell donor-specific anergy was 80 ± 10% (mean ± SD) (Table 1). The T_10_-cell product was constituted of 96 ± 4% (mean ± SD) CD4^+^ T cells; the remaining non-CD4^+^ T cells were donor-derived DC-10 cells that were irradiated and therefore dead or prone to die (Additional file 2). Coefficients of variation were high for both DC-10 and T_10_-cell yield, probably due to intrinsic differences among donors. The cut off anergy value for classifying T_10_ cells as anergic towards donor antigens was determined utilizing the “mean minus 2× SD” as statistical method [11]. This method was chosen based on our previous experience in the ALT-TEN trial [4]. Based on the eight T_10_-cell preparations generated from eight different donor pairs, the anergy cut off of T_10_ cells was 60%. Thus, T_10_-cell products for clinical-grade-compatible use will be considered anergic when the value is ≥60%.

**Fig. 4:**
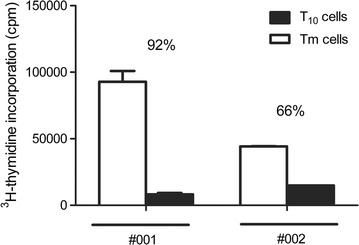
Protocol for T_10_-cell generation is reproducible upon up scaling from buffy coats to leukapheresis. CD4^+^ T cells purified from leukapheresis from two patients with kidney failure on dialysis were co-cultured with allogeneic DC-10 cells (generated from leukapheresis from two healthy donors) to generate T_10_ cells. Counts per min (cpm) of incorporated ^3^H-thymidine in the last 16–18 h of a 3-day co-culture of T_10_ or Tm cells with donor-mDC are shown. *Bars* represent mean value of cpm of T_10_ or Tm cells (in triplicates) generated from patient #001 and #002. Percentage of anergy of T_10_ cells is indicated over the *bars*

Based on our previous murine studies we plan to infuse a total of 2 × 10^6^ T_10_ cells/kg [12]. Given the DC-10 and T_10_-cell yield observed, buffy coats would fail to provide sufficient numbers of monocytes and CD4^+^ T cells to reach the required number of T_10_ cells. Up scaling from buffy coats to leukapheresis was therefore a necessary step [13]. As a proof-of-concept, leukapheresis from two healthy donors were used as sources of CD14^+^ cells for the generation of DC-10 cells. To generate T_10_ cells, leukapheresis from two patients with kidney failure on dialysis were used as sources of CD4^+^ T cells. This setting mimics exactly the clinical situation that we will face during the future clinical trial. The yield of T_10_ cells from both donor pairs surpassed the minimum number required for the planned infusions (2 × 10^6^/kg): 11 × 10^6^ cells/kg (#001) and 19 × 10^6^ cells/kg (#002). T_10_ cells displayed donor-specific anergy higher than 60% (Fig. 4). In addition to this, 3 more donor pairs were recruited in the study and T_10_ cells were successfully generated from leukapheresis in a GMP-compatible facility (Battaglia et al. manuscript in preparation), further proving protocol up scaling. These data demonstrate that the protocol for the generation of clinical-grade-compatible T_10_ cells defined by using buffy coats is also applicable to leukapheresis. Additionally, these data confirm our previous work tailoring the protocol to patients on dialysis [8]. A statistically significant positive correlation existed between the expression of the activation marker CD86 on DC-10 and T_10_-cell yield (Additional file 3). These data suggest that for DC-10 to generate antigen-specific T_10_ cells in vitro, they need to have an appropriate level of activation.

### In vitro characterization of the medicinal product

**Fig. 5:**
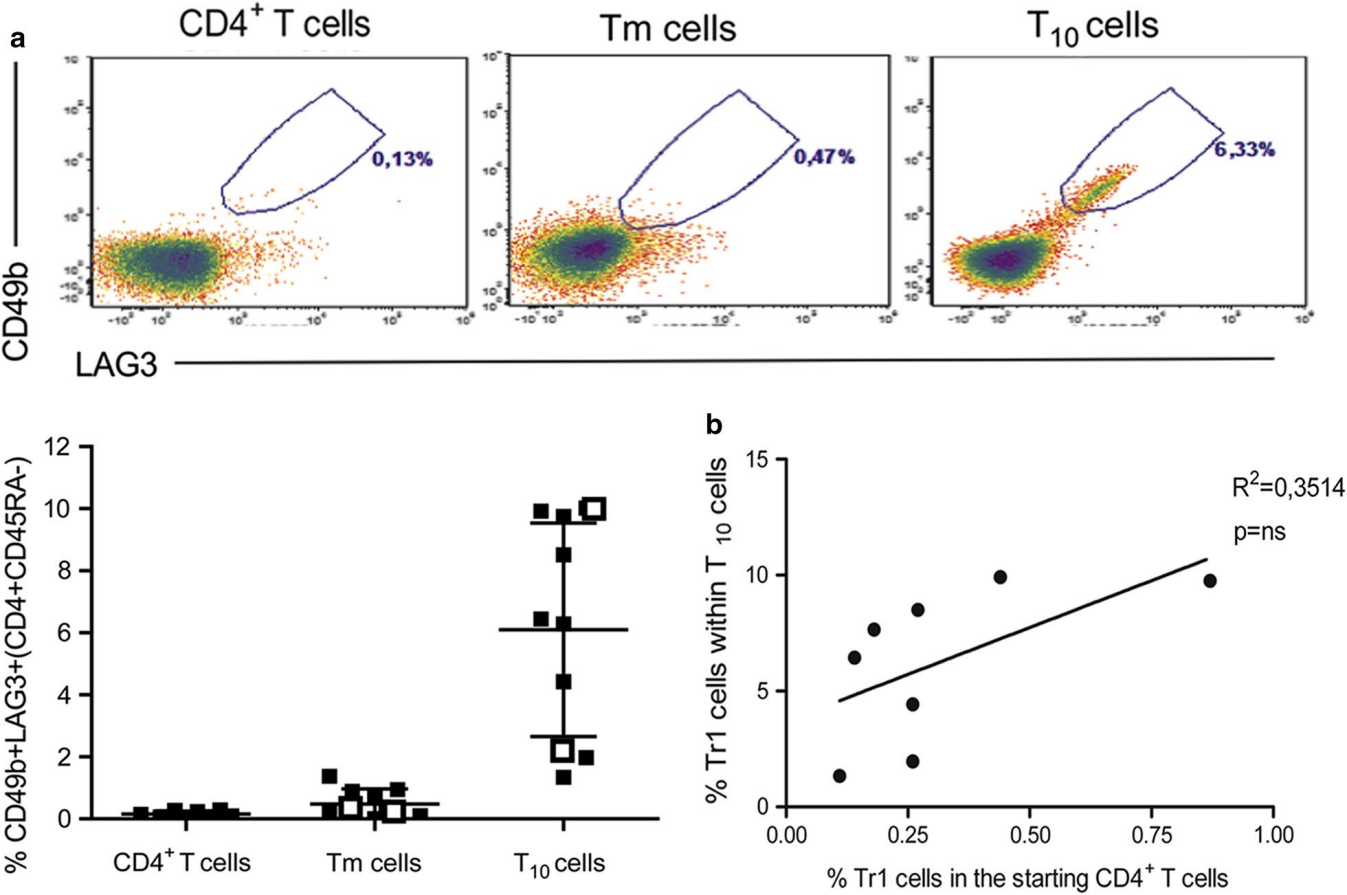
T_10_ cells are enriched in Tr1 cells. One representative flow cytometry staining (out of eight) of CD49b^+^LAG-3^+^ cells gated on CD4^+^CD45RA^−^ of CD4^+^ T cells, control Tm cells and T_10_ cells is shown (*upper panel* **a**). Percentages of CD49b^+^LAG-3^+^ (within CD4^+^CD45RA^−^) Tr1 cells are shown. Each *closed square* represents one experiment with buffy coats from healthy donors; each *open square* represents one experiment with leukapheresis from patients on dialysis. *Lines* represent mean value of each data set ± SD (*lower panel* **a**). Percentages of Tr1 cells in the starting CD4^+^ T cells are plotted against percentages of Tr1 cells within the corresponding T_10_ cells. *One dot* represents one experiment. *Line* represents linear regression (**b**)

Additional important functional features of T_10_ cells, that go beyond the development of the clinical-grade-compatible protocol, were also tested to better characterize the cells that will be infused into patients. As we had previously reported, the medicinal products generated in vitro with DC-10 are enriched in Tr1 cells and are comprised not only of Tr1 cells, but also CD4^+^ memory T cells that respond to nominal antigens [7]. To test the Tr1-cell content in the T_10_ cells generated with the clinical-grade-compatible protocol described above, the frequency of CD4^+^CD45RA^−^CD49b^+^LAG-3^+^ cells (i.e., Tr1 cells [14]) was tested by flow cytometry. T_10_ cells contained an average of 6 ± 3% (mean ± SD) Tr1 cells, while control Tm cells lacked Tr1 cells (Fig. 5a). Tr1-cell content in T_10_ cells generated from patients on dialysis using leukapheresis products was comparable to that in T_10_ cells generated from healthy donors using buffy coats (open versus closed squares, respectively Fig. 5a). The Tr1-cell content in T_10_ cells was irrespective of the frequency of Tr1 cells originally present in the starting CD4^+^ T-cell population, supporting the notion of de novo generation of donor-specific Tr1 cells rather than the expansion of circulating Tr1 cells (Fig. 5b).

**Fig. 6:**
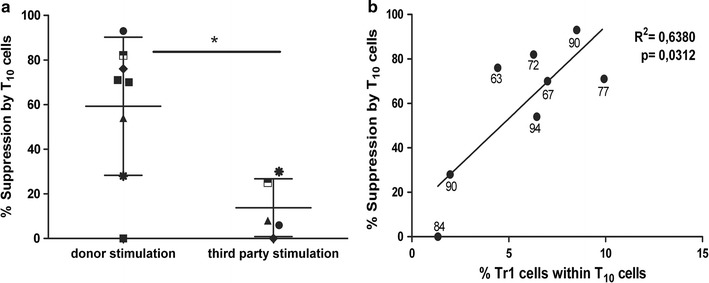
Donor-specific suppression of CD4^+^ T cell proliferation by T_10_ cells correlates with Tr1-cell content. CD4^+^ T cells were stimulated with donor mDC (or third party mDC—when numbers of T_10_ cells permitted) in the presence or absence of autologous T_10_ cells. Percentages of suppression by T_10_ cells are shown. *One dot* represents one experiment with buffy coats from healthy donors (*each symbol* depicts one donor and *different shapes* represent different donor pairs). *Lines* represent mean value of each data set ±SD (*p ≤ 0.05 Wilcoxon matched pairs test) (**a**). Percentages of Tr1-cell content within T_10_ cells is plotted against percentage of suppression by T_10_ cells. *One dot* represents one experiment. *Numbers* represent anergy levels measured on each T_10_-cell preparation. *Line* represents linear regression (**b**)

Treg-cell function is commonly tested in vitro by means of suppression assays [14, 15]. These assays are cumbersome and difficult to be used as standard tests in clinical-grade labs. However, the demonstration that T_10_ cells have in vitro suppressive activity further supports their potential efficacy in vivo in patients. The in vitro ability of T_10_ cells to suppress proliferation of autologous CD4^+^ T cells in response to donor-mDC was therefore tested. To prove donor-specific regulation, suppression of autologous CD4^+^ T cell responses towards third party allogeneic mDC was also tested when numbers of T_10_ cells permitted. Priority was given to anergy tests, being most relevant in the upcoming Phase 1 trial testing the safety of T_10_ cells. All, except one T_10_-cell preparation, suppressed in vitro proliferation of autologous CD4^+^ T cells in response to donor-mDC but not to third party-mDC stimulation proving their antigen-specific regulatory properties (Fig. 6a). Importantly, a positive correlation between Tr1-cell content and the suppressive capacity of T_10_ cells was observed thus further suggesting that the in vitro regulation of T_10_ cells is mediated by Tr1 cells (Fig. 6b). Interestingly, a strong correlation was observed when Tr1-cell content exceeded 5% indicating that T_10_ cell preparations—to have a good suppressive capacity—need to contain at least 5% of Tr1 cells: CD4^+^CD45RA^−^CD49b^+^LAG-3^+^. To note, one preparation resulted in T_10_ cells with no suppressive capacity and low Tr1-cell content. However, the anergy level of these T_10_ cells was 84%, suggesting that even with a low Tr1-cell content these cells remain anergic towards donor stimulation, hence complying with the safety requirement of this medicinal product.

**Fig. 7:**
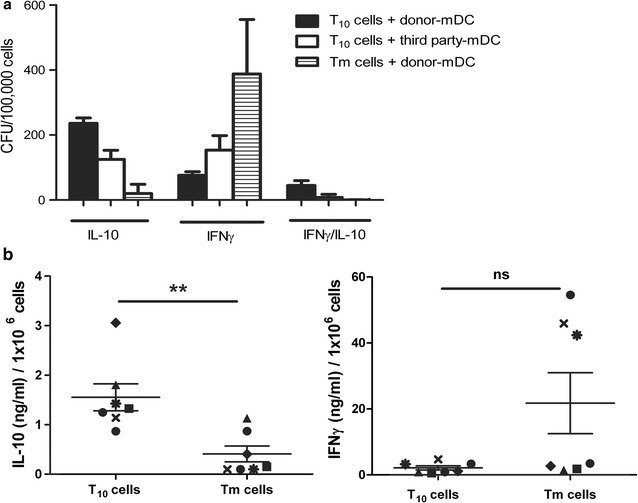
T_10_ cells produce more IL-10 and less IFNγ than control Tm cells in response to donor- but not third party-mDC stimulation. T_10_ or control Tm cells were stimulated with donor-mDC or third party-mDC. Cytokine-forming units (CFU) were detected by dual ELISPOT for IFNγ and/or IL-10. Number of CFU/100,000 cells is shown. *Bars* represent mean value of each dataset ±SD (n = 3 experiments) (**a**). T_10_ or control Tm cells were stimulated with donor-mDC. IL-10 and IFNγ measured in the supernatant 48 h after culture by ELISA is shown. *One dot* represents one experiment with buffy coats from healthy donors (*each symbol* depicts one donor and *different shapes* represent different donor pairs). *Lines* represent mean value of each data set ± SD (ns, **p < 0.005 Mann–Whitney test) (**b**)

A high number of IL-10-producing cells and a low number of IFNγ-producing cells in response to donor-mDC stimulation was detected by dual ELISPOT in T_10_ cells, as compared to those detected in control Tm cells (Fig. 7a). This was confirmed by the levels of IL-10 and IFNγ by ELISA in the supernatant of co-culture of T_10_ cells with donor-mDC (Fig. 7b).

T_10_ cells maintained a polyclonal TCR-Vβ repertoire proving lack of a skewed immune response in vitro (Additional file 4) supporting previous findings [4].

**Fig. 8:**
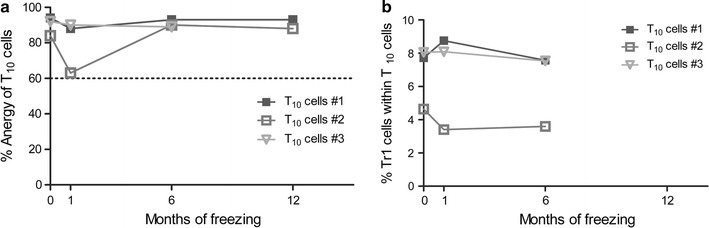
Freezing has little effect on donor-specific anergy and Tr1-cell content of T_10_ cells. T_10_ cells were thawed after 1, 6 or 12 months of freezing and immediately stimulated with donor-mDC. In parallel, flow cytometry staining for Tr1 cells was performed. Percentages of donor-specific anergy (**a**) and Tr1 cell content (**b**) are shown. *One symbol* represents one T_10_ cell preparation starting from buffy coats from healthy donors. *Lines* represent the time course of the percentage of anergy or Tr1-cell content of individual T_10_ cells thawed at different timepoints. *Dotted line* represents the set cutoff anergy level of 60%. Tr1-cell content 12 months after freezing was not available

We anticipate the infusion of T_10_ cells in patients only upon obtaining all safety data (i.e., quality controls and anergy values) on the medicinal product. Thus, cryopreservation of T_10_ cells is inevitable. However, not all cell products are suitable for freezing and thawing, requiring additional manipulation to restore their functionality upon thawing [16, 17]. We thus aimed at testing cell product viability and safety upon various cryopreservation time-points. All T_10_ cells had a viability ≥70% upon thawing (after 1, 6 or 12 months of cryopreservation) and preserved their Tr1-cell content (data not shown). All T_10_-cell preparations tested, remained anergic upon donor-mDC stimulation and preserved Tr1 content when thawed (Fig. 8). For limited cell number availability, the suppressive ability and/or the cytokine production profile of thawed products could not be tested. We gave priority to the anergy test, given that it provides a clear answer on the safety of thawed medicinal products. These data provide evidence that T_10_ cells can be cryopreserved up to 12 months without losing their viability, stability and donor-specific anergy upon thawing and do not require further manipulation prior to in vivo infusion. Similar data were obtained with the 3 GMP-grade generated medicinal products (Battaglia et al. manuscript in preparation).

### Gene signature of circulating Tr1 cells is transiently affected by immunosuppressive drugs

**Fig. 9:**
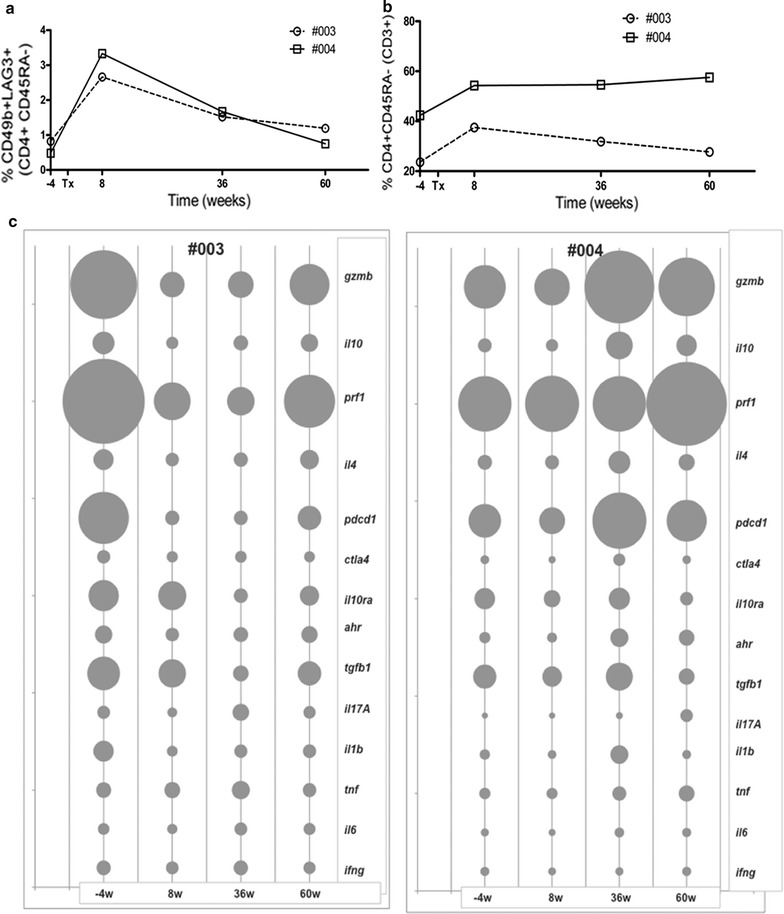
Gene signature of circulating Tr1 cells of two kidney transplant recipients is transiently affected by immmunosuppression. Percentages of Tr1 cells before (4 weeks pre-transplant) and after kidney transplantation (8, 36, 60 weeks) are shown. *One line* represents one patient (**a**). Percentages of circulating CD4^+^CD45RA^−^ T memory cells (within CD3^+^ T cells) in the two patients are shown (**b**). Transcript analysis of the indicated genes was performed on purified circulating Tr1 cells from patient #003 and #004. *Bubble chart* represents the gene expression signals for each patient over time: 4 weeks pre-transplant, 8-, 36- and 60-weeks post-transplant (normalized to housekeeping gene *hprt1*). Bubble size represents gene expression signal. *Gene symbols* are represented on the *right* (**c**)

The ONE Study trial with donor-specific Tr1 cells infused into patients undergoing kidney transplantation envisages the concomitant administration of immunosuppressive drugs [5]. Thus, the obvious question on administering cell therapy under active immunosuppression is the effect of the selected drugs on Tr1-cell viability and stability/function in vivo [18]. The ONE Study also included a parallel clinical trial with no cell therapy but standard immunosuppressive treatment, to be used as a reference group for the analysis of cell therapy trials (Additional file 5, detailing immunosuppressive regimen). We aimed at monitoring whether circulating endogenous Tr1 cells preserve their gene signature under standard immunosuppressive therapy: *il10* ^*hi*^ *, il4* ^*lo*^ *, il17* ^*lo*^ *, tgfβ* ^*hi/int*^ *, pd1* ^*hi*^ *, granzyme b* ^*hi*^, (reviewed in [1]). To this aim, circulating Tr1 cells of two patients enrolled in the Reference Group Trial at our clinical site were studied (Additional file 6 detailing immunosuppression dosages and trough levels). The frequency and gene expression profile of circulating Tr1 cells purified from patients after kidney transplant and under active immunosuppression (8, 36 and 60 weeks post-transplant) was compared to those of circulating Tr1 cells purified from the same patients before transplant (4 weeks pre-transplant) and in the absence of any drug treatment. Tr1 cells were detectable in the circulation and were increased in frequency and absolute numbers (Fig. 9a). Tr1-cell frequency peaked at 8 weeks post-transplant then returned to pre-transplant levels in both patients (Additional file 7). This increase was likely due to homeostatic proliferation after induction therapy, as observed in memory CD4^+^ T cells (Fig. 9b) [19]. Tr1 cells were isolated by flow cytometry-based cell sorting (Additional file 8 for gating strategy). The gene signature typical of Tr1 cells [14] was slightly diminished (patient #003) or remained unchanged (patient #004) at 8 weeks post-transplant and then returned to pre-transplant levels or heightened at 60 weeks post-transplant (in the two patients, respectively) (Fig. 9c).

These data suggest that Tr1 cells expand along with the CD4^+^ T-cell memory population and that the Tr1-cell tolerogenic gene expression profile remains stable even under active immunosuppressive treatment. Data are limited to two patients (being the only patients we enrolled in the reference group trial) but they attempted to dissect the Tr1-cell sensitivity to immunosuppression in vivo, a relevant concern that was—to our knowledge—never addressed before. Importantly, these data suggest that the best timing of ex vivo-generated Tr1-cell infusion could be right at the moment of transplant (to reduce inflammation and control alloreactivity) and around 36 weeks post-transplant, when the Tr1-cell signature starts to recover.

## Discussion

Optimization of a clinical-grade compatible protocol for generating donor-specific Tr1-enriched cell medicinal products is a pre-requisite for the planned clinical trial in kidney transplant recipients [5]. In this study we described a clinical-grade compatible protocol that enabled the production of donor-specific Tr1-cell enriched medicinal product (named T_10_ cells) by coculturing recipient CD4^+^ T cells with tolerogenic donor DC-10 in the presence of exogenous IL-10 for 10 days. The generated T_10_ cells are anergic and suppressive towards donor stimulation in vitro, maintain a stable function upon cryopreservation and are successfully produced in clinically sufficient amounts starting from leukapheresis from patients on dialysis. We also demonstrated that circulating Tr1 cells have a limited sensitivity (in terms of viability and gene expression profile) to standard immunosuppressive treatment in vivo.

Several hurdles are encountered when attempting to perform cell therapy clinical trials [18]. First, the protocol for generating the medicinal product needs to be clinical-grade. To that end, we surpassed all the obstacles. Second, a sufficient number of cells to be infused is a prerequisite. Up scaling to leukapheresis could, in some instances, represent a hurdle in terms of sample collection and protocol adaptability [20]. Here we proved that leukapheresis can be collected with no medical contra-indications from patients on dialysis and that both tolerogenic DC-10 and T_10_ cells can be generated from leukapheresis products.

Another key aspect is the definition of lab tests that ensure safety of the cell product. Within The ONE Study consortium, some groups are using polyclonal Tregs. We decided to invest in the donor-specific Tr1-cell-based therapy in an attempt to promote antigen-specific tolerance. However, the generation of T_10_ cells with donor-derived DC, although they are tolerogenic and well characterized [3, 7], contains an intrinsic risk of generating alloreactive T cells that, once infused, could be potentially risky for the patient leading to graft rejection. The ONE Study Cell Therapy Trials are feasibility and safety trials [5]. Thus, in vitro assays that prove medicinal-product safety are mandatory. Donor-specific anergy is an optimal assay to test T_10_-cell safety, while the suppression assay provides indications on the possible efficacy of Tr1 cells in vivo. Accordingly, T_10_ cells that show no suppressive capacity in vitro but retain donor-specific anergy are considered safe and therefore will be infused in patients participating in The ONE Study cell therapy trial at our institute.

Some groups working with FOXP3^+^ Tregs as cell therapy products, reported problems with cryopreservation, thus requiring—for instance—further cell manipulation upon thawing [16, 17]. The demonstration that T_10_ cells are stable and conserve their Tr1-cell content and donor-specific anergy properties upon cryopreservation, allows for flexibility in their preparation and feasibility for more than one infusion.

Whether infused T_10_ cells retain their viability and function in vivo under treatment with immunosuppressive drugs remains an important open question. One approach to address this issue was by testing the effect of immunosuppressive drugs in vitro on the ex vivo-expanded human FOXP3^+^ Tregs or by using humanized mouse models [21]. This showed detrimental dose-dependent effects of immunosuppressive treatments on viability and proliferative capacity while sparing the immunosuppressive function of FOXP3^+^ Tregs. We approached this issue by analyzing the frequency and the gene signature of circulating Tr1 cells collected from renal transplant recipients under active immunosuppressive treatment. Our data suggest that immunosuppressive drugs do not affect Tr1 cells since the cells remain in circulation and a transient change in the intensity of the gene signature is observed. Based on these data, we chose two different timings of T_10_-cell infusion to increase the chance of obtaining in vivo immune regulation. The first dose will be infused at the time of transplant (for Tr1-cell enrichment just around the transplant period). The second dose will be infused at 36 weeks post-transplant, timepoint in which the Tr1-cell signature is recovering.

Taken together, our results demonstrate the reproducibility of an optimized clinical-grade-compatible protocol for generating Tr1-enriched T_10_ cells. The necessary following steps for performing the trial in patients are underway.

## Conclusion

We describe the steps undertaken to achieve and validate a reproducible optimized clinical-grade compatible protocol capable of generating donor-specific Tr1 cells in sufficient numbers. Additionally, selecting the timing of infusion of Tr1 cells to patients under immunosuppression remains an open question. We provide data assessing the viability and gene signature of circulating Tr1 cells in the presence of active immunosuppression thus supporting our rationale for selecting the timing of the planned infusions. We believe that this study highlights the importance of optimizing and validating Tr1 cell manufacturing protocols to bring them closer to the bedside.

## Additional files

**Additional file 1.** Information on probe set used for defining the Tr1 cell signature.

**Additional file 2.** DC-10 cells represent the non-CD4+ cell population within the T_10_ cell preparations.

**Additional file 3.** Frequency of CD86+ DC-10 cells correlates with T_10_ cell yield.

**Additional file 4.** The TCR-Vβ repertoire of T_10_ cells is similar to that of the starting CD4+ T cells.

**Additional file 5.** Detailed immunosuppressive regimen as foreseen by The ONE Study Reference Trial.

**Additional file 6.** Dosages of immunosuppressive drugs per patient enrolled in The ONE Study reference group at each time point.

**Additional file 7.** Flow cytometry dot plots of circulating Tr1 cells in two kidney transplant recipients under active immunosuppression.

**Additional file 8.** Tr1 cell sorting gating strategy.

## Abbreviations

APC: antigen presenting cells
ATMP: advanced-therapy medicinal products
CV: coefficient of variation
DC: dendritic cells
DC-10: IL-10-producing dendritic cells
FBS: fetal bovine serum
GMP: good manufacturing practice
GvHD: graft versus host disease
HS: human serum
HSCT: hematopoietic stem cell transplantation
LPS: lipopolysaccharide
mDC: mature dendritic cells
MLR/DC-10: medicinal product constituted of donor PBMC anergized with irradiated host DC-10
PBMC: peripheral blood mononuclear cells
Tm: effector T mature cells
Tr1: T regulatory type 1
Treg: T regulatory
T_10_: medicinal product enriched in Tr1 cells
