# Supplementary material for: Generation of donor-specific Tr1 cells to be used after kidney transplantation and definition of the timing of their in vivo infusion in the presence of immunosuppression
Source: J Transl Med. 2017 Feb 21;15:40. doi: 10.1186/s12967-017-1133-8 (PMC5319067; doi:10.1186/s12967-017-1133-8)
Supplement: Supplementary file 1 — Additional file 1. Information on probe set used for defining the Tr1 cell signature. [file 12967_2017_1133_MOESM1_ESM.pdf]

Additional File 1. Information on probe set used for defining the Tr1 cell signature

Accession Number	Symbol	Other Names	Sequence Length	Probe Set Region	Specificity designed specifically to hybridize:
NM_004131	GZMB	HLP; CCPI; CGL1; CSPB; SECT; CGL-1; CSP-B; CTLA1; CTSGL1	941	425-916	human **Granzyme B**
NM_002190	IL17A	CTLA8; IL-17; IL17-A	1859	980-1731	human **IL17A**
NM_005041	PRF1	P1; PFP; HLPH2; MGC65093	2529	728-1180	human **Perforin1**, both transcript variants
NM_000572	IL10	CSIF; TGIF; IL-10; IL10A	1629	44-654	human **IL10**
NM_000576	IL1B	IL-1; IL1F2; IL1-BETA	1498	24-659	human **IL1β**
NM_000589	IL4	BSF1; IL-4; MGC79402	921	12-558	human **IL4**, both transcript variants
NM_005018	PDCD1	PD1; SLEB2; hPD-1; hPD-l	2115	255-796	human Programmed cell death protein 1 (**PD1**)
NM_005214	CTLA4	CD152; CTLA-4	2033	726-1307	human Cytotoxic T-lymphocyte-associated protein 4 (**CTLA4**)
NM_000194	HPRT1	HPRT; HGPRT	1435	673-1404	human Hypoxantine phosphoribosyltransferase 1 (**HPRT1**)
NM_000594	TNF	DIF; TNFA; TNFSF2; TNF-alpha	1669	812-1230	human Tumor necrosis factor alpha (**TNFα**)
NM_001558	IL10RA	IL10R; CDW210A; HIL-10R; IL-10R1	3672	255-653	human **IL10RA**, both transcript variants
NM_000600	IL6	HGF; HSF; BSF2; IL-6; IFNB2	1201	3-669	human **IL6**

NM_001621	AHR		6247	1289-1990	human Aryl hydrocarbon receptor (**AHR**)
NM_000660	TGFB1	CED; DPD1; TGF-beta	2217	1284-1649	human Transforming growth factor, beta 1 (**TGFb1**)
NM_000619	IFNG	IFG; IFI	1240	53-438	human interferon gamma (**IFNg)**
