# Supplementary material for: Generation of donor-specific Tr1 cells to be used after kidney transplantation and definition of the timing of their in vivo infusion in the presence of immunosuppression
Source: J Transl Med. 2017 Feb 21;15:40. doi: 10.1186/s12967-017-1133-8 (PMC5319067; doi:10.1186/s12967-017-1133-8)
Supplement: Supplementary file 2 — Additional file 2. DC-10 cells represent the non-CD4+ cell population within the T10 cell preparations. [file 12967_2017_1133_MOESM2_ESM.pdf]

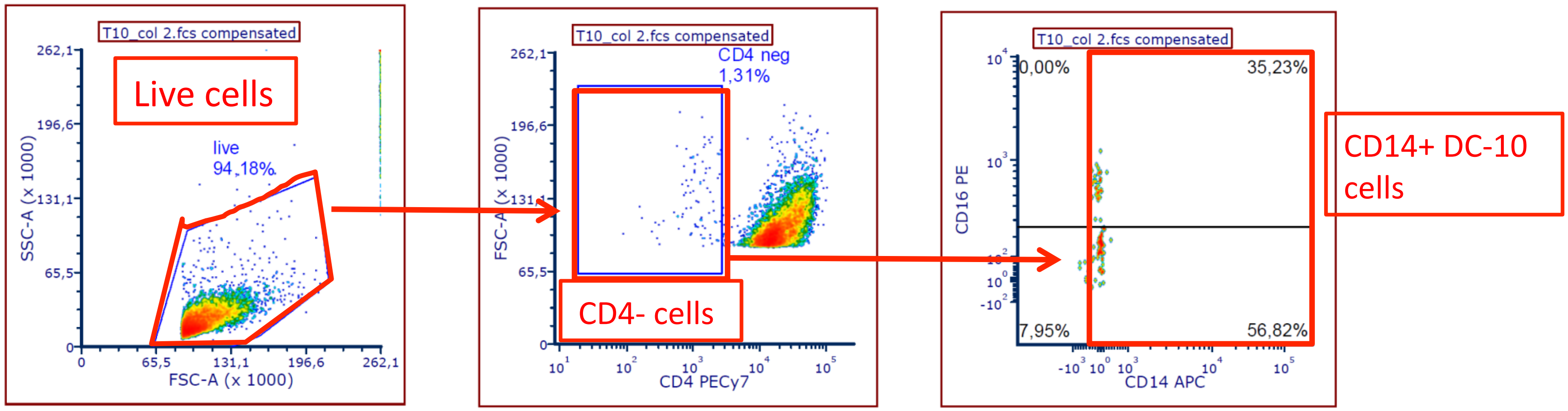

Additional File 3. DC-10 cells represent the non-CD4+ cell population within the T10 cell preparations. One representative dot plot of eight of T10 cells. Percentage of $CD4^{-}CD14^{+}$ cells is shown. Gating was done on live T10 cells.
