# Supplementary material for: Generation of donor-specific Tr1 cells to be used after kidney transplantation and definition of the timing of their in vivo infusion in the presence of immunosuppression
Source: J Transl Med. 2017 Feb 21;15:40. doi: 10.1186/s12967-017-1133-8 (PMC5319067; doi:10.1186/s12967-017-1133-8)
Supplement: Supplementary file 3 — Additional file 3. Frequency of CD86+ DC-10 cells correlates with T10 cell yield. [file 12967_2017_1133_MOESM3_ESM.pdf]

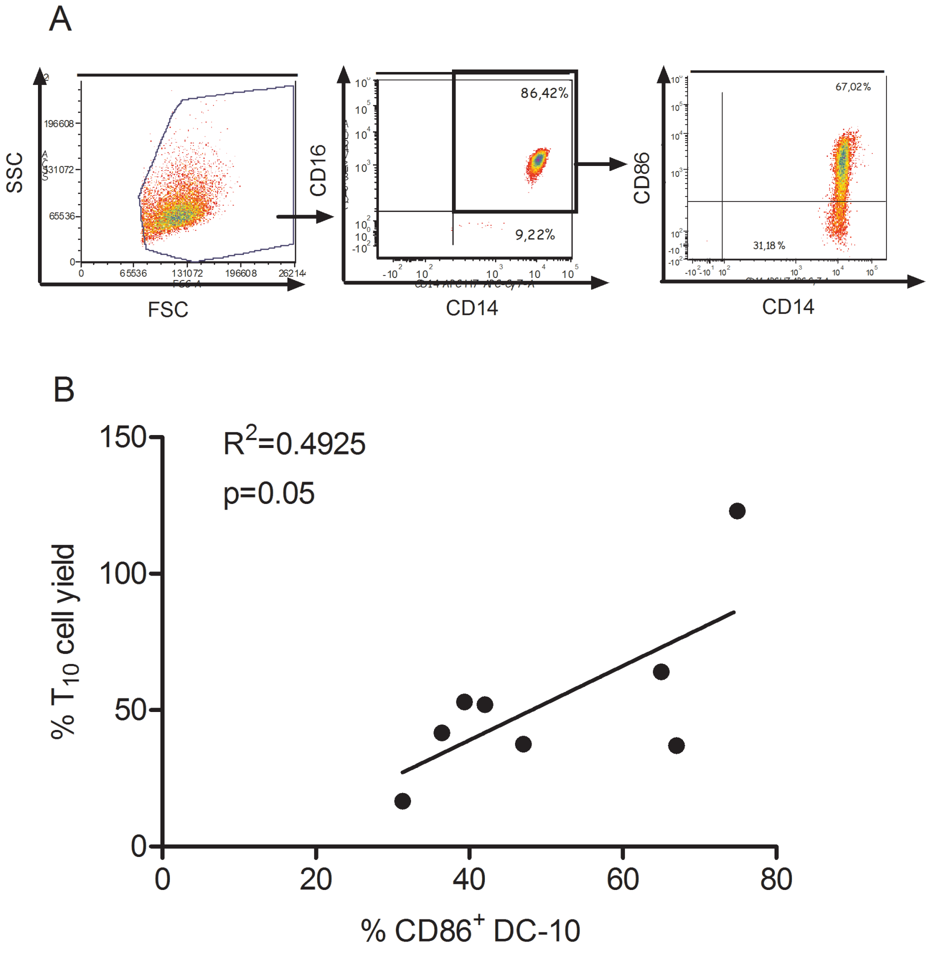

Additional File 4. Frequency of CD86$^+$ DC-10 cells correlates with T_{10} cell yield.

Phenotype of one representative preparation (out of eight) of DC-10 is shown (**A**). Percentages of CD86$^+$ DC-10 (CD14$^+$CD16$^+$) are plotted with yield of the corresponding T_{10} cells. Line represents linear regression (**B**).
