# Supplementary material for: Generation of donor-specific Tr1 cells to be used after kidney transplantation and definition of the timing of their in vivo infusion in the presence of immunosuppression
Source: J Transl Med. 2017 Feb 21;15:40. doi: 10.1186/s12967-017-1133-8 (PMC5319067; doi:10.1186/s12967-017-1133-8)
Supplement: Supplementary file 4 — Additional file 4. The TCR-Vβ repertoire of T10 cells is similar to that of the starting CD4+ T cells. [file 12967_2017_1133_MOESM4_ESM.pdf]

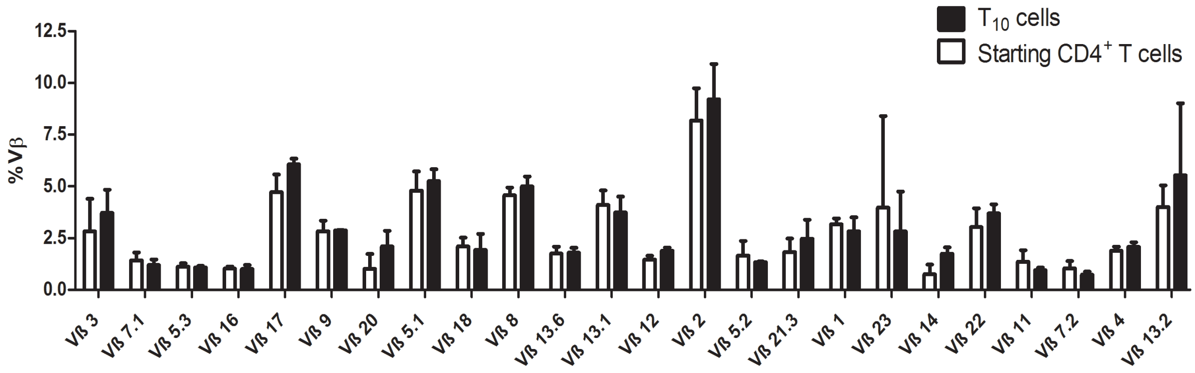

Additional File 5. The TCR-Vβ repertoire of T_{10} cells is similar to that of the starting $CD4^+$ T cells

Percentages of Vβ usage in T_{10} cells (■) and in the corresponding $CD4^+$ T cells before culture (□) are shown. Bars represent mean value of each dataset ± SD (n=5 T_{10}-cell preparations)
