# Supplementary material for: Generation of donor-specific Tr1 cells to be used after kidney transplantation and definition of the timing of their in vivo infusion in the presence of immunosuppression
Source: J Transl Med. 2017 Feb 21;15:40. doi: 10.1186/s12967-017-1133-8 (PMC5319067; doi:10.1186/s12967-017-1133-8)
Supplement: Supplementary file 5 — Additional file 5. Detailed immunosuppressive regimen as foreseen by The ONE Study Reference Trial. [file 12967_2017_1133_MOESM5_ESM.pdf]

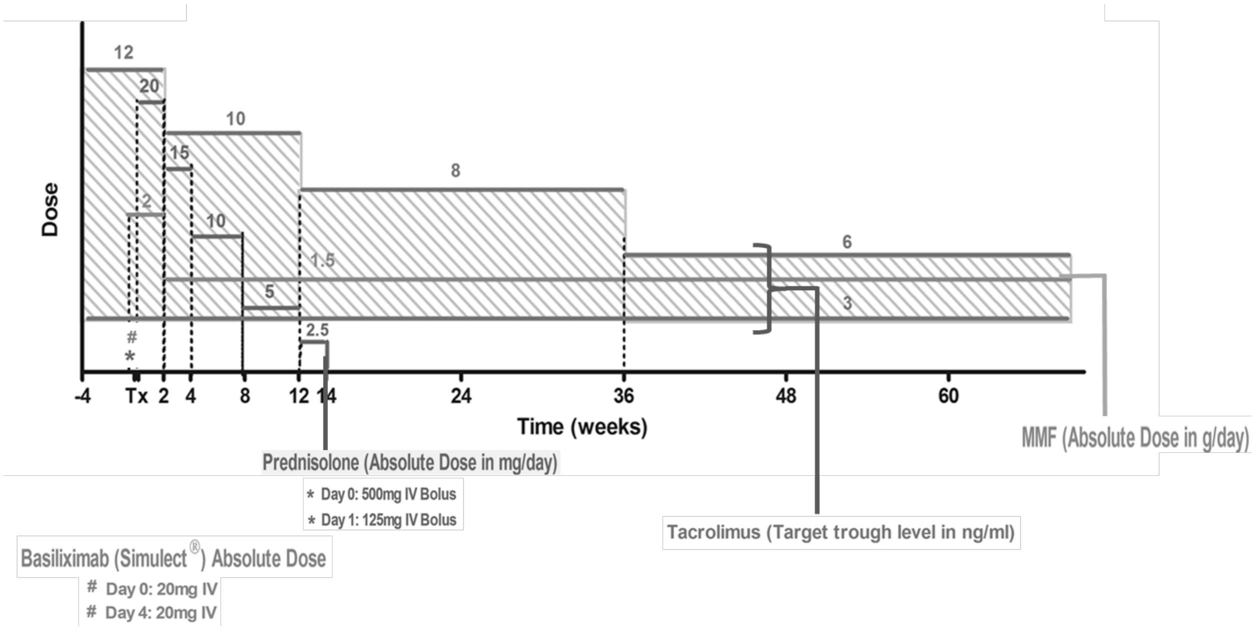

Additional File 6. Detailed immunosuppressive regimen as foreseen by The ONE Study Reference Trial
