# Supplementary material for: Generation of donor-specific Tr1 cells to be used after kidney transplantation and definition of the timing of their in vivo infusion in the presence of immunosuppression
Source: J Transl Med. 2017 Feb 21;15:40. doi: 10.1186/s12967-017-1133-8 (PMC5319067; doi:10.1186/s12967-017-1133-8)
Supplement: Supplementary file 6 — Additional file 6. Dosages of immunosuppressive drugs per patient enrolled in The ONE Study reference group at each time point. [file 12967_2017_1133_MOESM6_ESM.pdf]

Additional File 2. Dosages of immunosuppressive drugs per patient enrolled in The ONE Study reference group at each time point

	- 4 weeks PRE transplant		+ 8 weeks POST transplant		+ 36 weeks POST transplant		+ 60 weeks POST transplant	
	#003	#004	#003	#004	#003	#004	#003	#004
**Tacrolimus** trough levels (ng/ml)	-	-	10.6	10.6	9.9	5.8	9.1	4.3
target levels (ng/ml)	-		3-10		3-8		3-6	
**Prednisolone** (mg/day)	-		10		0		0	
**MMF** (g/day)	-		1.5		1.5		1.5	
**Basiliximab** (mg)	-		0		0		0	
