# Supplementary material for: Generation of donor-specific Tr1 cells to be used after kidney transplantation and definition of the timing of their in vivo infusion in the presence of immunosuppression
Source: J Transl Med. 2017 Feb 21;15:40. doi: 10.1186/s12967-017-1133-8 (PMC5319067; doi:10.1186/s12967-017-1133-8)
Supplement: Supplementary file 7 — Additional file 7. Flow cytometry dot plots of circulating Tr1 cells in two kidney transplant recipients under active immunosuppression. [file 12967_2017_1133_MOESM7_ESM.pdf]

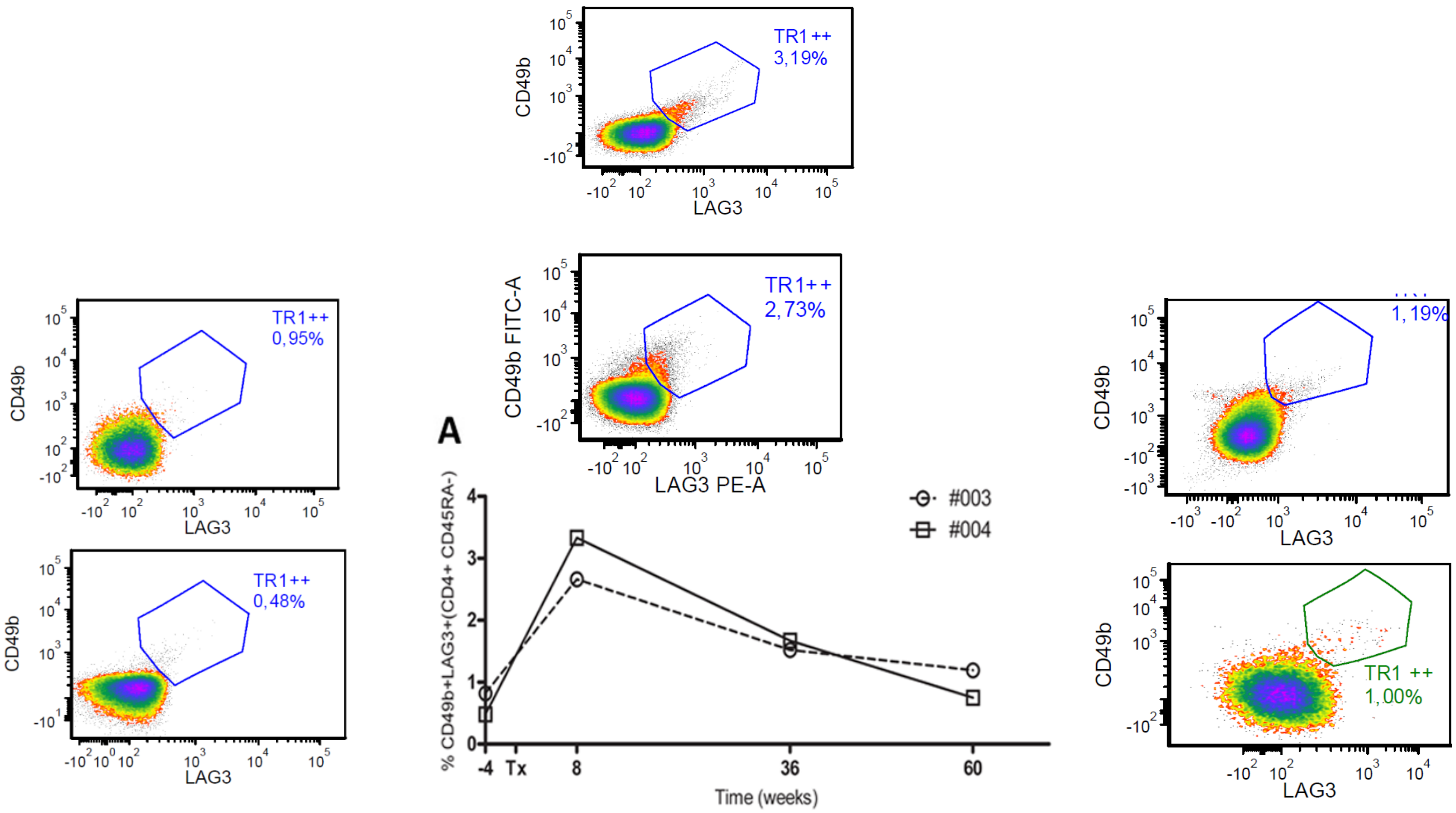

Additional File 7. Flow cytometry dot plots of circulating Tr1 cells in two kidney transplant recipients under active immunosuppression.
Percentages of Tr1 cells (CD49b$^+$LAG3$^+$) are shown pre- and post-transplantation. Gating was performed on live CD3$^+$CD4$^+$CD45RA$^-$ cells.
