# Supplementary material for: Generation of donor-specific Tr1 cells to be used after kidney transplantation and definition of the timing of their in vivo infusion in the presence of immunosuppression
Source: J Transl Med. 2017 Feb 21;15:40. doi: 10.1186/s12967-017-1133-8 (PMC5319067; doi:10.1186/s12967-017-1133-8)
Supplement: Supplementary file 8 — Additional file 8. Tr1 cell sorting gating strategy. [file 12967_2017_1133_MOESM8_ESM.pdf]

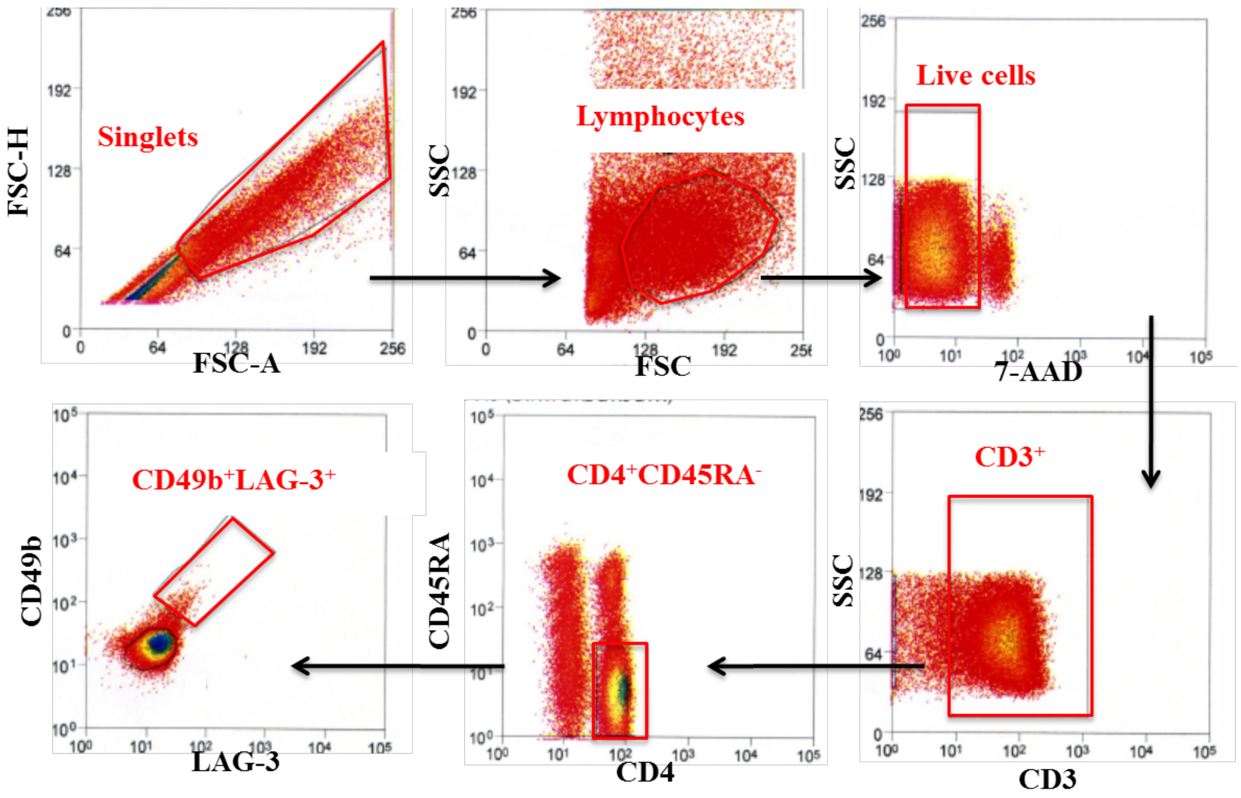


**Additional File 8. Tr1 cell sorting gating strategy**

Tr1 cells were sorted from thawed PBMC. Gating strategy is shown: from singlets (FSC-A, FSC-H), to lymphocytes (FSC, SSC), live cells (SSC, $7AAD^-$), T cells (SSC, $CD3^+$), memory $CD4^+$ T cells ($CD4^+CD45RA^-$), and Tr1 cells ($CD49b^+LAG3^+$).
